# Ancient DNA analysis of food remains in human dental calculus from the Edo period, Japan

**DOI:** 10.1371/journal.pone.0226654

**Published:** 2020-03-04

**Authors:** Rikai Sawafuji, Aiko Saso, Wataru Suda, Masahira Hattori, Shintaroh Ueda

**Affiliations:** 1 Department of Human Biology and Anatomy, Graduate School of Medicine, University of the Ryukyus, Nakagami, Okinawa, Japan; 2 Department of Biological Sciences, Graduate School of Science, The University of Tokyo, Bunkyo-ku, Tokyo, Japan; 3 The University Museum, The University of Tokyo, Bunkyo-ku, Tokyo, Japan; 4 Department of Physical Therapy, Faculty of Rehabilitation, Niigata University of Health and Welfare, Kita-ku, Niigata, Japan; 5 RIKEN Center for Integrative Medical Sciences (IMS), Laboratory for Microbiome Sciences, Yokohama, Kanagawa, Japan; 6 Cooperative Major in Advanced Health Science, Graduate School of Advanced Science and Engineering, Waseda University, Okubo Shinjuku-ku, Tokyo, Japan; 7 School of Medicine, Hangzhou Normal University, Hangzhou, Zhejiang, People’s Republic of China; University of Helsinki, FINLAND

## Abstract

Although there are many methods for reconstructing diets of the past, detailed taxon identification is still challenging, and most plants hardly remain at a site. In this study, we applied DNA metabarcoding to dental calculus of premodern Japan for the taxonomic identification of food items. DNA was extracted from 13 human dental calculi from the Unko-in site (18th–19th century) of the Edo period, Japan. Polymerase chain reaction (PCR) and sequencing were performed using a primer set specific to the genus *Oryza* because rice (*Oryza sativa*) was a staple food and this was the only member of this genus present in Japan at that time. DNA metabarcoding targeting plants, animals (meat and fish), and fungi were also carried out to investigate dietary diversity. We detected amplified products of the genus *Oryza* from more than half of the samples using PCR and Sanger sequencing. DNA metabarcoding enabled us to identify taxa of plants and fungi, although taxa of animals were not detected, except human. Most of the plant taxonomic groups (family/genus level) are present in Japan and include candidate species consumed as food at that time, as confirmed by historical literature. The other groups featured in the lifestyle of Edo people, such as for medicinal purposes and tobacco. The results indicate that plant DNA analysis from calculus provides information about food diversity and lifestyle habits from the past and can complement other analytical methods such as microparticle analysis and stable isotope analysis.

## Introduction

Ancient diets have been revealed by multiple methods such as analysis of plant and faunal remains at sites, stable isotope analysis, organic residue analysis of pottery, dental microwear analysis, and morphological analysis of microparticles such as phytoliths and starch grains. Starch grains and phytoliths within ancient calculus (calcified dental plaque) are direct evidence of food items and have revealed dietary habits [1,2], the spread of domesticated plants [3–5], cooking [6], and other usages of teeth [7–9]. Although the conventional methods are powerful and have been applied to many studies, there are some challenges. For example, taxonomic identification of food at the species or genus level is often difficult, and sometimes the criteria used for assessing this are not completely objective. Moreover, analysis of tissues that hardly remain at a site (e.g., leaves, roots, and rhizomes) is almost impossible.

Food DNA analysis of dental calculus has the potential to overcome these limitations. Ancient calculus is one of the richest known sources of ancient biomolecules in the archeological record [10–12]. DNA analysis enables detailed taxon identification of plants and animals. In fact, Warinner et al. (2014) [13] and Weyrich et al. (2017) [14] detected plant and animal DNA possibly derived from consumed food, but some challenges with this approach still remain. The efficacy of food DNA analysis of dental calculus has not been adequately validated, and there is a need to improve it as a methodology to analyze the food consumed in the past. Previous studies detected plant and animal DNA from calculus using shotgun sequencing, but the proportion of plant/animal DNA was quite low. For example, Warinner et al. (2014) [13] reported that DNA within calculus is dominated by bacterial DNA (>99%), with a very small proportion derived from other sources including food DNA. The composition of DNA within calculus was reported to be as follows: 0.002% for animals, 0.005% for fungi, and 0.008% for plants [13]. Weyrich et al. (2017) [14] reported that Neanderthal samples contained 0.27% eukaryotic sequences. With such a small proportion, the cost of food DNA analysis is enormous and run-to-run carryover could be a serious problem. Approximately 0.002% carryover contamination (i.e., contamination from previous sequencing runs) was reported using an Illumina sequencer [15,16]. This implies that the risk of misidentification of carryover contamination as food is relatively high because each taxon of food has almost the same proportion of carryover contamination when applying shotgun DNA sequencing to dental calculus.

There is also the matter of databases [17]. The level of completeness of reference databases varies by genomic region, which may cause misidentification of taxa. For example, the numbers of species/genera with complete chloroplast genomes were 2,255/1,172 in the National Center for Biotechnology Information (NCBI) RefSeq database (as per the release of 15 September 2017); meanwhile, the approximate numbers of species/genera with depositions of the trnL region of chloroplasts were 72,587/11,037 (downloaded from GenBank on 25 October 2017). Thus, DNA metabarcoding using regions for which abundant information is contained in databases would be more suitable for taxon identification than shotgun sequencing.

In this study, we analyzed food DNA in ancient calculus using DNA metabarcoding analysis, in order to overcome the above-mentioned problems. DNA metabarcoding is a method using a standardized DNA region as a tag for accurate and simultaneous identification of many taxa [18,19]. It is often used in the field of ecology for characterizing diet content [20,21] and has been applied to ancient DNA analysis for taxon identification of bulk bone [22–24], permafrost [25], and lake sediment [26,27]. By applying this method to calculus samples of Edo people, we investigated whether ancient calculus contains a variety of candidate food DNA including that from staple food.

## Materials and methods

### Sample sites

The Unko-in site is the former graveyard of the Unko-in temple at Fukagawa, Tokyo (S1 Table) [28]. The excavation was conducted in 1955, and more than 200 skeletons were excavated. In terms of the chronological age of the materials, they were dated to the latter half of the Edo period (from the 18th to the 19th century), as determined from the accompanying cultural finds and historical documents about the temple [29,30]. The people buried at the temple were townspeople of Edo City, as inferred from their graves and the posthumous Buddhist names (*kaimyo* in Japanese) written on them [31]. The skeletons are housed at the University Museum, the University of Tokyo (UMUT).

### Sampling

**Fig 1:**
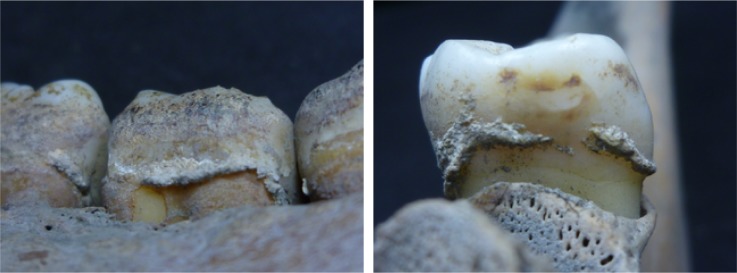
Close-up views of dental calculus on the teeth from the sampled individuals of the Unko-in site.

Supragingival calculi were collected from the teeth of 14 human adult skeletons using a sterilized dental explorer (YDM Corporation), the identity of which was confirmed by morphological observation (Fig 1). Sampling was performed at UMUT. For each individual, we combined the calculi from multiple teeth. Calculus from each individual was collected separately into a 1.5 ml DNA LoBind tube (Eppendorf). For comparison, a soil sample from the mandibular foramen was also collected and subjected to the same analysis as the calculus, as a control. Masks, nitrile gloves, hairnets, and laboratory coats were worn throughout the process.

### General equipment

DNA extractions and library preparations were carried out in a dedicated ancient DNA laboratory that was physically separated from the one where polymerase chain reaction (PCR) cycling was conducted. Masks, nitrile gloves, hairnets, and laboratory coats were worn throughout the process and were replaced regularly. Neither latex gloves nor woolen clothes were used, in order to avoid contamination [32,33]. Items such as tubes and pestles were UV-irradiated before use. Workspaces were wiped with 5% bleach or DNA Away (Thermo Fisher Scientific) and rinsed with 80% ethanol. Laboratory equipment was UV-irradiated, treated with 5% bleach or DNA Away, and rinsed with 80% ethanol. Filtered pipette tips were used in all steps. All DNA extractions and PCR reactions included negative controls.

### Confirmation of oral bacteria

To verify the presence of oral bacteria in dental calculus, we performed 16S rRNA analysis. This research was carried out as a collaboration between bioarchaeologists and bacteriologists. The bacteriologists have established the protocol of 16S rRNA analysis from modern calculus, so we applied it to 16S rRNA analysis. DNA was extracted from one calculus sample (wn2016-F41) from Unko-in site using 1 ml of 0.5 M EDTA and the Fast DNA spin kit for soil (MP Biomedicals), followed by ethanol precipitation.

PCR was performed using the primers 27Fmod (5′-AGRGTTTGATYMTGGCTCAG-3′) and 338R (5′-TGCTGCCTCCCGTAGGAGT-3′) [34]. The amplification was carried out in 1 × Ex Taq PCR buffer (50 μl), with dNTPs (2.5 mM), Ex Taq polymerase (Takara), each primer (10 μM), and 8.0 μl of template DNA. The cycling profile included an initial denaturation step at 96°C for 2 min; followed by 25 cycles of 96°C for 30 s, 55°C for 45 s, and 72°C for 1 min; and final extension at 72°C for 10 min. Negative controls were included in the PCR amplification. PCR amplicons were purified using the Agencourt AMPure XP kit (Beckman Coulter Genomics), quantified using the Quant-iT PicoGreen dsDNA Assay Kit (Life Technologies), and then sequenced using either 454 GS FLX Titanium or 454 GS JUNIOR (Roche Applied Science). Data analysis was performed as previously described [35]: We used the custom combined database from three publicly available databases: Ribosomal Database Project (RDP), CORE (http://microbiome.osu.edu/), and a reference genome sequence database obtained from the NCBI FTP site (ftp://ftp.ncbi.nih.gov/genbank/). Reads were clustered at 96% similarity. We analyzed the resulting taxonomic data by scoring the assigned genera to the categories of “Oral” or “Other” as inferred by the presence or absence of each genus in the Human Oral Microbiome Database [36], as used in Ziesemer et al. (2015) [37].

### DNA extraction

The extraction procedure was a modified version of that reported by Dabney et al. (2013) [38] and Ozga et al. (2016) [39]. Dental calculus samples were UV-irradiated for 1 min on each side. After washing twice with molecular-grade water, samples were resuspended for several hours at 55°C in 500 μl of a buffer of 0.5 M EDTA. Samples were then homogenized using each sterile plastic pestle (AS ONE). The digestion step was performed by adding 400 μl of a buffer of 0.5 M EDTA and 10% proteinase K, followed by 8–12 h of digestion at 55°C and 24 h of digestion at 37°C on a rotator. Remaining precipitation was then pelleted by centrifugation in a bench-top centrifuge for 15 min at maximum speed. The supernatant was added to 13 ml of binding buffer, which contained final concentrations of 5 M guanidine hydrochloride, 40% (vol/vol) isopropanol, 0.05% Tween-20, and 90 mM sodium acetate (pH 5.2). A binding apparatus was constructed by forcefully fitting an extension reservoir removed from a Zymo-Spin V column (Zymo Research) into a MinElute silica spin column (Qiagen). The extension reservoir-MinElute assembly was then placed into a 50 ml falcon tube. The 14 ml solution containing binding buffer and the extraction supernatant was then poured into the extension reservoir, and the falcon tube cap was secured. The binding apparatus was centrifuged for 5 min at 1,600 *g*. The extension reservoir-MinElute column was removed from the falcon tube and placed into a clean 2 ml collection tube. The extension reservoir was then removed, and two washing steps were performed by adding 720 μl of binding buffer to the MinElute column, centrifuging on a bench-top centrifuge, and discarding the flow-through. Then, two washing steps were performed by adding 720 μl of PE buffer (Qiagen) to the MinElute column, centrifuging on a bench-top centrifuge, and discarding the flow-through. The MinElute column was dry-spun for 1 min at maximum speed (15,000 rpm) in a bench-top centrifuge and then placed in a fresh 1.5 ml DNA LoBind tube (Eppendorf). For elution, 15 μl of EB buffer was pipetted onto the silica membrane and after 5 min of incubation was collected by centrifugation for 1 min at maximum speed. This step was repeated for a total of 30 μl of DNA extract. One microliter of each extract was quantified using a Qubit High Sensitivity dsDNA assay (Life Technologies).

### PCR of the genus *Oryza* (*atpE* gene)

For the detection of rice DNA in calculus samples, PCR was performed using *atpE* gene primers, the sequences of which were specific to the genus *Oryza atpE* gene: atpE_F1 (5′-CGTATTCTCAAGGGACCCATATCT-3′) and atpE_R1 (5′-GCCAAATTGGCGTATTACCAA-3′) [40]. This pair of primers were selected on the basis of the following criteria: (1) The amplified sequence is *Oryza*-specific, as confirmed by BLAST (blastn-megablast) [41]. (2) The amplified sequence is shorter than 100 bp (expected size: 70 bp) because of the highly fragmented nature of ancient DNA [42]. (3) The sequence is present in the chloroplast, mitochondrial, and nuclear genome, because they are usually present in high copy numbers and the sequence is expected to be easily amplified. (4) The primer sequences are largely divergent from sequences of bacteria, archaea, and fungi, to avoid nonspecific amplification.

Each PCR reaction mixture contained 22.6 μl of molecular-grade water, 4 μl of 10 × PCR buffer, 1.0 U AmpliTaq Gold DNA Polymerase (Applied Biosystems), 3.2 μl of MgCl_2_ (25 mM), 4 μl of dNTPs (2 mM), 2 μl of each primer (10 μM), and 2.0 μl of DNA template (5 ng/μl) for a total volume of 40 μl. PCR thermal cycling conditions were as follows: 9 min at 95°C; 40 cycles of 30 s at 95°C, 30 s at 50°C, and 30 s at 72°C; and, finally, 7 min at 72°C. Negative controls (water) were included in the PCR amplification, in order to verify the PCR efficiency and to detect contamination, if any. We performed positive control experiments in a separate laboratory (modern DNA lab).

After the PCR amplification, 4–9 μl of PCR solution was loaded on MCE-202 MultiNA (Shimadzu), a capillary microchip electrophoresis system for DNA analysis. For sequencing, second PCR was performed using 1 μl of product from the first PCR with the same conditions as before, but for 10 cycles. Nucleotide sequences of the PCR products were obtained and analyzed in an ABI 3730xl DNA Analyzer (Applied Biosystems) or Applied Biosystems 3130xl Genetic Analyzer by the Fasmac sequencing service (Fasmac). Sequencing was conducted in both directions.

### DNA metabarcoding

DNA extracts and extraction blank controls were amplified using four DNA metabarcoding markers: *trn*L intron (P6 loop of the chloroplast *trn*L intron) for plants, 12S rRNA (two different primer sets) for vertebrates/teleosts, and ITS1 for fungi [43,44].

Analysis of faunal food residues by DNA metabarcoding requires one more step. There is a problem that samples such as feces and calculus are often enriched with host DNA (i.e., humans in this study), so the result of PCR amplification could be dominated by host DNA rather than by prey DNA [45]. To overcome this problem, a “blocking primer” has been used to reduce the amplification of host DNA. This primer preferentially binds to host DNA and is modified so that it does not prime amplification. There are various types of blocking primer, and the most effective and common type is the primer that overlaps with the 3′ end of the universal primer but extends into host-specific sequence and is modified with a C3 spacer (three hydrocarbons) at the 3′ end [46]. This C3 spacer prevents elongation during PCR, so prey DNA is preferentially amplified.

As for animal DNA metabarcoding, it is expected that human DNA would be amplified predominantly when targeting animal DNA, so we used human blocking primer sets for DNA metabarcoding with 12S rRNA primer sets. The sequences of primers used for DNA metabarcoding are listed in Table 1.

**Table 1:** List of primers used for DNA metabarcoding in this study.

Reference	(Taberlet et al., 2007)	(Taberlet et al., 2007)	(Riaz et al., 2011)	(Riaz et al., 2011)	(De Barba et al., 2014)	(Valentini et al., 2016)	(Valentini et al., 2016)	(Valentini et al., 2016)	(T. J. White, Bruns, Lee, & Taylor, 1990)	(Epp et al., 2012)
Average product length (bp)	50		100			100			250	
Primer sequence (5′-3′)	GGGCAATCCTGAGCCAA	TTGAGTCTCTGCACCTATC	TAGAACAGGCTCCTCTAG	TTAGATACCCCACTATGC	CTATGCTTAGCCCTAAACCTCAACAGTTAAATCAACAAAACTGCT-C3	ACACCGCCCGTCACTCT	CTTCCGGTACACTTACCATG	ACCCTCCTCAAGTATACTTCAAAGGAC-C3	GGAAGTAAAAGTCGTAACAAGG	CAAGAGATCCGTTGTTGAAAGTT
Primer name	trnL-g	trnL-h	12SV5_F	12SV5_R	12SV5_F_blk_hum	teleo_F	teleo_R	teleo_F_blk_hum	ITS5	5.8S_fungi
DNA region	*trn*L intron (P6 loop)		12S rRNA			12S rRNA			ITS	
Target taxon	Plants		Vertebrates			Teleosts			Fungi	

All primers were modified to include a subset of Nextera XT (Illumina) adapters. Each PCR reaction mixture of the first PCR contained 11.38 μl of molecular-grade water, 2.5 μl of 10 × PCR buffer, 0.6 U AmpliTaq Gold DNA Polymerase (Applied Biosystems), 2 μl of MgCl_2_ (25 mM), 2.5 μl of dNTPs (2 mM), 1.25 μl of each primer (10 μM), and 4 μl of DNA template (5 ng/μl) for a total volume of 25 μl. PCR thermal cycling conditions were as follows: 9 min at 95°C; 40 cycles of 30 s at 95°C, 30 s at 50°C, and 30 s at 72°C; and finally 7 min at 72°C. PCR products were purified using Agencourt AMPure XP kit (Beckman Coulter Genomics) and quantified using the HS dsDNA Qubit Assay on a Qubit 2.0 Fluorometer (Life Technologies).

The second-round PCR (second PCR) used the first PCR products as a template. Each PCR reaction mixture of the first PCR contained 25 μl of 2 × KAPA HiFi HotStart ReadyMix (KAPA Biosystems), 5 μl of each Nextera XT Index Primer 1, 5 μl of each Nextera XT Index Primer 2, and 15 μl of the first PCR products as a template, for a total volume of 50 μl. PCR products were purified using Agencourt AMPure XP kit (Beckman Coulter Genomics), quantified using the HS dsDNA Qubit Assay on a Qubit 2.0 Fluorometer (Life Technologies), and visualized using a High Sensitivity DNA Assay Chip kit on a Bioanalyzer 2100 (Agilent). Samples with less than 0.5 ng/μl DNA were discarded.

Purified PCR products were pooled to equimolar concentration (4 nM). Five microliters of the pool library were denatured with 5 μl of fresh 0.1 N NaOH. Including HT1 buffer (provided by Illumina), the denatured library was diluted to a final concentration of 4–8 pM. Here 5% PhiX DNA spike-in control was added to improve data quality of low-diversity samples such as PCR amplicons (Carpenter et al., 2013; Miya et al., 2015). The libraries of paired-end reads were sequenced with Illumina MiSeq (Illumina Inc.) using MiSeq Reagent Kit Nano and Micro v2 (2 × 150 bp). The raw fastq files obtained in the present study were deposited in the DNA Data Bank of Japan (DDBJ) Sequence Read Archive (accession number: DRA009382).

### Data analysis

Nextera XT adapters were removed from paired-end reads using cutadapt v.1.11 [47]. Trimmed paired-end reads were then merged using the *illuminapairedend* tool in OBITOOLS [48]. Unmerged reads were removed using *obigrep*. Sequences with counts ≤10 were removed using *obiuniq*, *obistat*, and *obigrep*. Each sequence was then assigned the status of “head,” “internal,” or “singleton” using *obiclean*. Since all sequences labeled “internal” probably correspond to PCR substitutions and indel errors, only “head” and “singleton” sequences were used for sequential taxonomic assignment. All primer sequences were removed because mutations may be inserted in the process of PCR amplification. Taxonomic assignments were identified using blastn-megablast on the NCBI website (https://blast.ncbi.nlm.nih.gov/Blast.cgi) with the database of the NCBI nucleotide collection (nr/nt) [41]. For plant (*trn*L primer sets) and animal (12S rRNA primer sets) DNA metabarcoding, taxa with 100% query coverage and 100% identity were accepted. For fungal (ITS primer sets) DNA metabarcoding, taxa with 99% identity were accepted. Identification was determined from the genus to the order level from accepted taxa.

The taxa detected from soil samples were removed from among those used for the analysis of calculus samples. Functional annotations of fungal genera were determined by the descriptions of Tedersoo et al. (2014) [49]. All necessary permits were obtained for the described study, which complied with all relevant regulations.

## Results

### DNA extraction

We extracted DNA successfully from 13 samples out of 14 samples. DNA extraction yields are shown in Table 2. The total amount of DNA was 206–1,650 ng and normalized yields of DNA were 13–90 ng/mg calculus, which is far more than for DNA extracted from bone and dentine [50]. Warinner et al. (2015) [10] and Mann et al. (2018) [51] showed higher or equal yields of DNA from ancient calculus, so the yields in this research are not unreasonably high. The proportion of the "Oral" category is 73% in Extraction 1 and 97% in Extraction 2, respectively. We also confirmed the presence of oral bacteria such as *Streptococcus parasanguinis* and *Streptococcus salivarius* (S2 Table).

**Table 2:** Ancient dental calculus information and DNA extraction yields.

Individuals	Sex	Sample (mg)	Total DNA (ng)	Normalized DNA yield (ng/mg)
wn2016-F01	Female	11	345	31.4
wn2016-F04	Female	16	206	12.9
wn2016-F07	Male	20	627	31.4
wn2016-F08	Female	16	1434	89.6
wn2016-F10	Male	29	690	23.8
wn2016-F12	Female	35	1410	40.3
wn2016-F23	Male	19	333	17.5
wn2016-F24	Male	17	699	41.1
wn2016-F37	Female	29	1476	50.9
wn2016-F39	Male	33	930	28.2
wn2016-F41	Male	43	1650	38.4
wn2016-F43	Female	23	1050	45.7
wn2016-F44	Female	12	1038	86.5

### Plants

**Fig 2:**
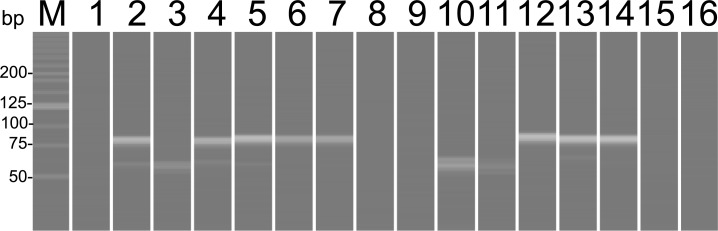
PCR amplification products of dental calculus using *Oryza atpE* gene primer sets. M, molecular weight markers; 1, wn2016-F01; 2, wn2016-F04; 3, wn2016-F07; 4, wn2016-F08; 5, wn2016-F10; 6, wn2016-F12; 7, wn2016-F23; 8, negative control; 9, wn2016-F24; 10, wn2016-F37; 11, wn2016-F39; 12, wn2016-F41; 13, wn2016-F43; 14, wn2016-F44; 15, soil from wn2016-F39; 16, negative control.

We examined whether DNA of the genus *Oryza*, which includes rice species (*Oryza sativa*), was detected from ancient calculus in Edo people. This is because rice was a staple food of people living in Edo City and is likely to be detected from such calculus. DNA amplification of *Oryza* was detected in eight out of 13 calculus samples by PCR, as shown in Fig 2. The sequences of the PCR products were identified as the genus *Oryza* (E value = 2.0 × 10^−26^), which included a cultivated rice taxon (*O*. *sativa*). There was no significant difference between the sexes (Fisher's exact test, *p* = 0.59).

Next, we investigated whether other genera of plants could be detected from ancient calculus samples using DNA metabarcoding analysis (shown in Table 3). For DNA metabarcoding, the P6 loop of the chloroplast *trn*L intron was amplified using the primers trnL-g and trnL-h [52], as shown in Table 1. After identification with Blast, seven taxa were confirmed at the family level and 10 taxa were confirmed at the genus level from six samples in total, as shown in Table 4. The number of reads identified per sample is shown in S3 Table. Sequences of the family Fagaceae were detected from four individuals, and sequences of the family Poaceae and the genera *Lactuca*, *Celtis*, and *Oryza* were detected from two individuals. Other taxa were detected from one individual. The sequence of *Ginkgo biloba* was detected from samples of soil, calculus, and blank, and we excluded this sequence from further analyses. The sequence of the family Araceae was also detected from a blank sample.

**Table 3:** Results of DNA metabarcoding.

Individuals	Plants (trnL)	Vertebrates (12S rRNA)	Teleosts (12S rRNA)	Fungi (ITS)
wn2016-F04	✓✓	-	-	*
wn2016-F08	-	*Homo*	-	✓✓
wn2016-F10	✓✓	-	-	✓✓
wn2016-F12	✓✓	-	-	✓✓
wn2016-F23	-	-	-	-
wn2016-F41	✓✓	-	-	-
wn2016-F43	✓✓	-	-	✓✓
wn2016-F44	✓✓	-	-	✓✓
Soil	✓✓	-	-	✓✓

Experiments that did not produce any identified sequences are shown by a hyphen. An asterisk indicates that the experiment was not performed. In the wn2016-F08 sample, human DNA was amplified with the primer set for vertebrates, which is represented as *Homo*. The soil sample was obtained from mandibular foramen of wn2016-F39.

**Table 4:** Plant taxa identified using *trn*L primer set.

	Taxon	Candidate food species/candidate usage	Individuals	Reference^2^
Family	Apiaceae	Carrot	wn2016-F41	(Miyazaki, 1697)
	Polygonaceae	Water pepper	wn2016-F44	(Miyazaki, 1697)
	Cucurbitaceae	Watermelon, Pumpkin	wn2016-F44	(Miyazaki, 1697)
	Cupressaceae	material of chopstick or oil	wn2016-F44	(Kaibara, 1709) (Hozumi, 1693)
	Dipterocarpaceae	Natural medicine (borneol)	wn2016-F44	(Terajima, 1712)
	Poaceae	Barley, Wheat	wn2016-F12, wn2016-F43	(Miyazaki, 1697)
	Fagaceae	Japanese chestnut	wn2016-F01, wn2016-F12, wn2016-F43, wn2016-F44	(Miyazaki, 1697)
Genus	*Perilla*	Shiso	wn2016-F12	(Miyazaki, 1697)
	*Plantago*	Chinese plantain	wn2016-F44	(Terajima, 1712)
	*Camellia*	Tea	wn2016-F44	(Miyazaki, 1697)
	*Brassica*^*1*^	Chinese mustard	wn2016-F01	(Miyazaki, 1697)
	*Raphanus*^*1*^	Radish	wn2016-F41	(Miyazaki, 1697)
	*Nicotiana*^*1*^	Tobacco	wn2016-F10	(Miyazaki, 1697)
	*Allium*	Japanese bunching onion, Asian chives	wn2016-F01	(Miyazaki, 1697)
	*Lactuca*	Lettuce	wn2016-F01, wn2016-F44	(Miyazaki, 1697)
	*Celtis*	Chinese hackberry	wn2016-F01, wn2016-F43	(Terajima, 1712)
	*Oryza*	Rice	wn2016-F01, wn2016-F44	(Miyazaki, 1697)

^1^Genus exists in Japan out of multiple genus candidates (Iwatsuki et al., 1995; Makino, 2008).

^2^Historical literature describes each species as being used for food or other purposes.

### Animals and fungi

We also investigated whether animal DNA could be detected from ancient calculus samples. We used “12SV5” primer pairs for the amplification of vertebrate DNA and “teleo” primer pairs for the amplification of teleosts [53,54]. The results are shown in Table 3. No animal taxon except human was detected from the calculus samples. Human DNA was detected from one sample. No sequence was detected from blank samples.

With regard to fungi, 3 taxa were confirmed at the order level, 4 taxa at the family level, and 12 at the genus level, from five calculus samples in total (S4 Table). We could not determine the origin of these fungi. Some fungi might be derived from plant pathogens, but it should be noted that some fungi might be derived from soil or mold which would occur during storage in the museum.

## Discussion

The purpose of this study was to assess the potential utility of ancient calculus DNA as a source of dietary evidence. Indeed, we demonstrated that plant DNA can be extracted from ancient calculus and identified at the family or genus level. Most of the identified taxa included species that were described as food in the historical literature of that time (Table 4). For example, lettuce (*Lactuca sativa*) was described as the food plant *chisha* (in Japanese) in *Nogyo Zensho*, published in the Edo period [55].

In particular, we detected *Oryza* sequences by both genus-specific PCR and DNA metabarcoding. Amplification was observed from more than half of the calculus samples in genus-specific PCR. No amplification products could be obtained from the soil and negative samples, so these results suggest that the *Oryza* DNA detected from calculus samples is derived from rice consumed as food.

The diet of townspeople in Edo City has been investigated by analyzing historical literature and performing stable isotope analysis. Historical studies suggested that the diet of the Edo townspeople was mainly composed of rice and vegetables, accompanied by fish [56]. Stable isotope analysis revealed that C3-based terrestrial food (e.g., rice and vegetables), freshwater fish, and marine fish were their main sources of dietary protein [57]. It is consistent with these reports that DNA of the genus *Oryza* and taxa including a variety of vegetables was detected from calculus samples in this study, although fish DNA was not detected. DNA metabarcoding of ancient calculus samples thus appears to be a promising approach for screening the diversity of plant food from the past.

### Plants

For the genus *Oryza*, there is a difference between the results of PCR obtained with the *Oryza*-specific primer set and DNA metabarcoding with the *trn*L primer set. PCR products were detected in more than half of the samples (eight out of 13 calculus samples) when using the specific primer set targeting sequences on mitochondria, chloroplasts, and the nucleus. On the other hand, a sequence of the genus *Oryza* was detected from only two out of eight samples when using the chloroplast *trn*L primer set for DNA metabarcoding analysis. This can be explained by the number of genome regions in which the sequences exist. The numbers of mitochondrial and chloroplast DNA copies contained in each organelle range from 10 to more than 100. Moreover, multiple copies of both organelles are contained in a cell, so the sequences present in both mitochondrial and chloroplast genomes are very easy to amplify by PCR. We selected this specific primer set because the sequences exist in multiple regions, including two sites of the mitochondrial genome, one site of the chloroplast genome, and eight sites of the nuclear genome. This can explain why the sequences of the genus *Oryza* were detected in the experiment with the specific primers much more than in that with the *trn*L primer set.

DNA of the genus *Allium* was detected in this study. Darbyshire & Henry (1981) [58] reported that no starch was detected from *Allium* species, although fructans were present. Shibutani (2015) [59] also reported that many Japanese *Allium* species produced a relatively small number of starch grains, so it seems to be quite difficult to identify *Allium* species by starch grain analysis of archeological materials. Our results indicate that plant DNA analysis from calculus enables us to identify even plants hardly remain at a site or plants that produce little starch.

Taxa that are difficult to interpret as food were also detected from the calculus samples (Table 4). Wild species of the genus *Nicotiana* did not exist in Japan [60,61], and only cultivated species for making tobacco were present at that time. Smoking was common among the townspeople in Edo City [62], so it seems reasonable that this taxon was detected from the calculus samples, although a pipe was normally used and chewing tobacco has not been recorded.

The source of the plant of the Cupressaceae family may be timber or resin. There is historical literature that Edo people used wood of Cupressaceae as chopsticks or tooth picking [63]. Hozumi (1693) [64] reported that they used Japanese cypress resin as a painkiller of toothache.

The detection of plants of the Dipterocarpaceae family is surprising because these trees are well-known constituents of Asian rainforests [65] and do not inhabit Japan. Therefore, this cannot be explained without the existence of trade. Dipterocarpaceae species which may have been imported into Japan and have Japanese names are *Dryobalanops aromatica*, *Shorea robusta* and *Vatica mangachapoi* [66]. All of these can’t grow in Japan.

One plausible explanation is that this was derived from impurities of borneol (*ryunou* in Japanese), chemical compounds extracted from *Dryobalanops aromatica* (*ryunou-jyu* in Japanese), belonging to the family Dipterocarpaceae [67]. Borneol is one of the traditional herbal medicines and was commonly used as a component of tooth powder in the Edo period. Tooth powder in Edo City was made mainly from *bousyu-zuna* (fine sand from Chiba Prefecture), flavored with borneol and clove [67,68]. Toothbrushing was common among the townspeople of Edo City, so DNA of Dipterocarpaceae could have been derived from borneol in tooth powder.

Detecting evidence of ancient oral hygiene activities is often difficult. It has been considered that interproximal wear grooves on fossil teeth could have been caused by tooth picking in order to extract food stuck between teeth, though a number of other hypotheses for the cause of the grooves have been proposed [9,69]. Previous studies analyzed microfossils and chemical compounds from dental calculus and suggested the performance of oral care or the use of a toothbrush or toothpick [70,71]. We found DNA that could be derived from tooth powder from ancient dental calculus, though more research is necessary to determine the origin of the Dipterocarpaceae DNA.

### Animals and fungi

In this study, we used a blocking primer of human DNA, but meat or fish DNA could not be identified from the calculus samples. Previous studies reported that blocking primer inhibits the amplification of target DNA in some cases [45,72]. We reduced the concentration of human blocking primers considering those studies. However, there is a possibility that blocking primers worked as an inhibitor of the PCR reaction because no animal DNA was detected. This is troublesome because human DNA would be amplified without blocking primers. In fact, human DNA was amplified even with blocking primers (Table 3). This dilemma is difficult to resolve, and study using modern calculus is needed to confirm this interpretation and improve the method.

Fungal DNA was also detected from the calculus samples (S4 Table). It is interesting that the functional group of many taxa was white rot (wood-decaying fungi). The proportion of white-rot fungi in soil is quite small (<1%) [49], and we excluded taxa that were detected from soil samples. Other identified taxa such as *Alternaria* and *Clonostachys* are plant pathogens, which might have been derived from plants used as food [14].

### Advantages and limitations of this method

The advantages of food DNA analysis of calculus include the possibility of performing genus/species-level identification. Species-level identification was not performed in this study, but it would theoretically be possible if specific primers were used. This method also enables us to detect plant species that hardly remain at a site (e.g., leaves, roots, and rhizomes) and can complement other methods such as stable isotope and microparticle analyses.

Food DNA in calculus can directly reveal the diversity of food consumed in the past, including in prehistoric times. This analysis can also be used to investigate the existence of trade. In this study, evidence of trade was presented from the identification of plants of the family Dipterocarpaceae. A previous study pointed out that materials within calculus include not only food but also nonfood items relevant to oral hygiene practices [73]. For example, the use of medicinal herbs has been discussed in studies of dental calculus using DNA, microparticle, and chemical analyses [14,70,74]. We found DNA that could be derived from tooth powder from an archeological material for the first time. Combining multiple methods such as proteomics [13,75], stable isotope analysis [76,77], and microparticle analysis [78,79] should lead to a deeper understanding of various facets of human life in the past.

With regard to the merits of DNA metabarcoding, it is cost-effective and suited to population analysis. It is also compatible with screening for food DNA. In the field of sedimentary ancient DNA (*sed*aDNA), DNA metabarcoding is a common method for analyzing vegetation and fauna [80,81], and its use may also spread to ancient calculus studies for dietary analysis.

There are also some limitations on DNA metabarcoding. The amplification might be skewed towards preferential amplification of certain taxa by using universal primers, which means that other taxa remain undetected [82]. Indeed, Ziesemer et al. (2015) [37] also pointed out that there is systematic amplification bias when applying 16S rRNA analysis to ancient calculus because of PCR product size. Therefore, DNA metabarcoding result might not cover all taxa that are present in a sample. As for ancient DNA, the sequence length is relatively short, so it is very difficult to design species-specific primers. Therefore, this research provided only candidates of species. We took no PCR replication, but it is desirable for reliable identification in the future.

There is a possibility that the nuclear genes or genome can be analyzed from the food debris of calculus. It was reported that high-throughput sequencing technologies are not suitable for analyzing the genome from charred archeobotanicals [83]. Calculus is not charred, so it seems likely that nuclear sequences could be obtained from it. If a taxon of interest is detected by DNA metabarcoding, one can expand the analysis to more specific research, for example, examining nuclear genes or correlations with oral microbiota.

## Supporting information

S1 Table Information of the specimens. (XLSX)

S2 Table The number of reads of each bacteria detected from dental calculus. (XLSX)

S3 Table The number of reads identified per sample. (XLSX)

S4 Table Fungal taxa identified using ITS primer set. (XLSX)

S1 Fig (TIF)

## References

[1] Henry AG, Henry AG, Brooks AS, Brooks AS, Piperno DR, Piperno DR. Microfossils in calculus demonstrate consumption of plants and cooked foods in Neanderthal diets (Shanidar III, Iraq; Spy I and II, Belgium). Proceedings of the National Academy of Sciences 2011;108:486–91. 10.1073/pnas.1016868108 PMC3021051 21187393

[2] Power RC, Salazar-García DC, Straus LG, González Morales MR, Henry AG. Microremains from El Mirón Cave human dental calculus suggest a mixed plant–animal subsistence economy during the Magdalenian in Northern Iberia. J Archaeol Sci 2015;60:39–46.

[3] Piperno DR, Dillehay TD. Starch grains on human teeth reveal early broad crop diet in northern Peru. Proceedings of the National Academy of Sciences 2008;105:19622–7. 10.1073/pnas.0808752105 PMC2604935 19066222

[4] Madella M, García-Granero JJ, Out WA, Ryan P, Usai D. Microbotanical evidence of domestic cereals in Africa 7000 years ago. PLoS One 2014;9:e110177 10.1371/journal.pone.0110177 25338096 PMC4206403

[5] Cristiani E, Radini A, Edinborough M, Bori D an. Dental calculus reveals Mesolithic foragers in the Balkans consumed domesticated plant foods. Proceedings of the National Academy of Sciences 2016;113:10298–303. 10.1073/pnas.1603477113 PMC5027412 27573829

[6] Barton H, Torrence R. Cooking up recipes for ancient starch: assessing current methodologies and looking to the future. J Archaeol Sci 2015;56:194–201.

[7] Blatt SH, Redmond BG, Cassman V, Sciulli PW. Dirty teeth and ancient trade: Evidence of cotton fibres in human dental calculus from Late Woodland, Ohio. International Journal of Osteoarchaeology 2010;21:669–78.

[8] Hardy K, Radini A, Buckley S, Blasco R, Copeland L, Burjachs F, et al Diet and environment 1.2 million years ago revealed through analysis of dental calculus from Europe’s oldest hominin at Sima del Elefante, Spain. Naturwissenschaften 2017;104:2 10.1007/s00114-016-1420-x 27981368

[9] Ungar PS, Grine FE, Teaford MF, Pérez-Pérez A. A review of interproximal wear grooves on fossil hominin teeth with new evidence from Olduvai Gorge. Arch Oral Biol 2001;46:285–92. 10.1016/s0003-9969(00)00128-x 11269862

[10] Warinner C, Speller C, Collins MJ. A new era in palaeomicrobiology: prospects for ancient dental calculus as a long-term record of the human oral microbiome. Philos Trans R Soc Lond B Biol Sci 2015;370:20130376–20130376. 10.1098/rstb.2013.0376 25487328 PMC4275884

[11] Hendy J, Warinner C, Bouwman A, Collins MJ, Fiddyment S, Fischer R, et al Proteomic evidence of dietary sources in ancient dental calculus. Proc Biol Sci 2018;285 10.1098/rspb.2018.0977 30051838 PMC6083251

[12] Jersie-Christensen RR, Lanigan LT, Lyon D, Mackie M, Belstrøm D, Kelstrup CD, et al Quantitative metaproteomics of medieval dental calculus reveals individual oral health status. Nat Commun 2018;9:4744 10.1038/s41467-018-07148-3 30459334 PMC6246597

[13] Warinner C, Rodrigues J o. FM, Vyas R, Trachsel C, Shved N, Grossmann J, et al Pathogens and host immunity in the ancient human oral cavity. Nat Genet 2014;46:336–44. 10.1038/ng.2906 24562188 PMC3969750

[14] Weyrich LS, Duchêne S, Soubrier J, Arriola L, Llamas B, Breen J, et al Neanderthal behaviour, diet, and disease inferred from ancient DNA in dental calculus. Nature 2017;544:357–61. 10.1038/nature21674 28273061

[15] Illumina. Reducing Run-to-Run Carryover on the MiSeq Using Dilute Sodium Hypochlorite Solution. 2013.

[16] Bartram J, Mountjoy E, Brooks T, Hancock J, Williamson H, Wright G, et al Accurate sample assignment in a multiplexed, ultrasensitive, high-throughput sequencing assay for minimal residual disease. J Mol Diagn 2016;18:494–506. 10.1016/j.jmoldx.2016.02.008 27183494

[17] Parducci L, Bennett KD, Ficetola GF, Alsos IG, Suyama Y, Wood JR, et al Ancient plant DNA in lake sediments. New Phytol 2017;214:924–42. 10.1111/nph.14470 28370025

[18] Pompanon F, Deagle BE, Symondson WOC, Jarman SN. Who is eating what: diet assessment using next generation sequencing. Mol Ecol 2012;21:1931–50. 10.1111/j.1365-294X.2011.05403.x 22171763

[19] Taberlet P, Coissac E, Pompanon F, Brochmann C, Willerslev E. Towards next-generation biodiversity assessment using DNA metabarcoding. Mol Ecol 2012;21:2045–50. 10.1111/j.1365-294X.2012.05470.x 22486824

[20] De Barba M, Miquel C, Mercier C, Rioux D. DNA metabarcoding multiplexing and validation of data accuracy for diet assessment: application to omnivorous diet. Mol Ecol Resour 2014;14:306–23. 10.1111/1755-0998.12188 24128180

[21] Shehzad W, Riaz T, Nawaz MA, Miquel C, Poillot C, Shah SA, et al Carnivore diet analysis based on next-generation sequencing: application to the leopard cat (Prionailurus bengalensis) in Pakistan. Mol Ecol 2012;21:1951–65. 10.1111/j.1365-294X.2011.05424.x 22250784

[22] Grealy A, Douglass K, Haile J, Bruwer C, Gough C, Bunce M. Tropical ancient DNA from bulk archaeological fish bone reveals the subsistence practices of a historic coastal community in southwest Madagascar. J Archaeol Sci 2016;75:82–8.

[23] Grealy A, Macken A, Allentoft ME, Rawlence NJ, Reed E, Bunce M. An assessment of ancient DNA preservation in Holocene-Pleistocene fossil bone excavated from the world heritage Naracoorte Caves, South Australia. J Quat Sci 2016;31:33–45.

[24] Murray DC, Haile J, Dortch J, White NE, Haouchar D, Bellgard MI, et al Scrapheap challenge: a novel bulk-bone metabarcoding method to investigate ancient DNA in faunal assemblages. Sci Rep 2013;3:3371 10.1038/srep03371 24288018 PMC3842778

[25] Willerslev E, Davison J, Moora M, Zobel M, Coissac E, Edwards ME, et al Fifty thousand years of Arctic vegetation and megafaunal diet. Nature 2014;506:47 10.1038/nature12921 24499916

[26] Parducci L, Jørgensen T, Tollefsrud MM, Elverland E, Alm T, Fontana SL, et al Glacial survival of boreal trees in northern Scandinavia. Science 2012;335:1083–6. 10.1126/science.1216043 22383845

[27] Epp LS, Gussarova G, Boessenkool S, Olsen J, Haile J, Schrøder-Nielsen A, et al Lake sediment multi-taxon DNA from North Greenland records early post-glacial appearance of vascular plants and accurately tracks environmental changes. Quat Sci Rev 2015;117:152–63.

[28] TAISEI ENGINEERING Co. L. Unko-in iseki [in Japanese]. Koto-ku kyoiku iinkai; 2010.

[29] Dodo Y. Non-metric traits in the Japanese crania of the Edo period. Bull Natl Sci Mus 1975;1:41–54.

[30] Suzuki H, Sakura H, Ehara A. Fukagawa Unko-in shutsudo no Edo jidaijin toukotsu ni tsuite [in Japanese]. Proceedings of the Joint Meeting of the Anthropological Society of Nippon and the Japanese Society of Ethnology, 11th Session 1957:102–5.

[31] Suzuki H. Nihonjin no hone [in Japanese]. Iwanami shoten; 1963.

[32] Velsko IM, Warinner C. Bioarchaeology of the Human Microbiome. Bioarchaeology International 2017;1:86–99.

[33] Soto M, Inwood J, Clarke S, Crowther A, Covelli D, Favreau J, et al Structural characterization and decontamination of dental calculus for ancient starch research. Archaeol Anthropol Sci 2019;11:4847–72.

[34] Kim S-W, Suda W, Kim S, Oshima K, Fukuda S, Ohno H, et al Robustness of gut microbiota of healthy adults in response to probiotic intervention revealed by high-throughput pyrosequencing. DNA Res 2013;20:241–53. 10.1093/dnares/dst006 23571675 PMC3686430

[35] Said HS, Suda W, Nakagome S, Chinen H, Oshima K, Kim S, et al Dysbiosis of salivary microbiota in inflammatory bowel disease and its association with oral immunological biomarkers. DNA Res 2014;21:15–25. 10.1093/dnares/dst037 24013298 PMC3925391

[36] Chen T, Yu W-H, Izard J, Baranova OV, Lakshmanan A, Dewhirst FE. The Human Oral Microbiome Database: a web accessible resource for investigating oral microbe taxonomic and genomic information. Database 2010;2010:baq013 10.1093/database/baq013 20624719 PMC2911848

[37] Ziesemer KA, Mann AE, Sankaranarayanan K, Schroeder H, Ozga AT, Brandt BW, et al Intrinsic challenges in ancient microbiome reconstruction using 16S rRNA gene amplification. Sci Rep 2015;5:16498 10.1038/srep16498 26563586 PMC4643231

[38] Dabney J, Knapp M, Glocke I, Gansauge MT, Weihmann A, Nickel B, et al Complete mitochondrial genome sequence of a Middle Pleistocene cave bear reconstructed from ultrashort DNA fragments. Proceedings of the National Academy of Sciences 2013;110:15758–63. 10.1073/pnas.1314445110 PMC3785785 24019490

[39] Ozga AT, Nieves-Colón MA, Honap TP, Sankaranarayanan K, Hofman CA, Milner GR, et al Successful enrichment and recovery of whole mitochondrial genomes from ancient human dental calculus. Am J Phys Anthropol 2016;160:220–8. 10.1002/ajpa.22960 26989998 PMC4866892

[40] Tozawa Y, Teraishi M, Sasaki T, Sonoike K, Nishiyama Y, Itaya M, et al The plastid sigma factor SIG1 maintains photosystem I activity via regulated expression of the psaA operon in rice chloroplasts. Plant J 2007;52:124–32. 10.1111/j.1365-313X.2007.03216.x 17651366

[41] Altschul SF, Gish W, Miller W, Myers EW, Lipman DJ. Basic local alignment search tool. J Mol Biol 1990;215:403–10. 10.1016/S0022-2836(05)80360-2 2231712

[42] Carpenter ML, Buenrostro JD, Valdiosera C, Schroeder H, Allentoft ME, Sikora M, et al Pulling out the 1%: whole-genome capture for the targeted enrichment of ancient DNA sequencing libraries. Am J Hum Genet 2013;93:852–64. 10.1016/j.ajhg.2013.10.002 24568772 PMC3824117

[43] White TJ, Bruns T, Lee S, Taylor J. Amplification and direct sequencing of fungal ribosomal RNA genes for phylogenetics. PCR Protocols, Elsevier; 1990, p. 315–22.

[44] Epp LS, Boessenkool S, Haile J, Esposito A, Erséus C, Gusarov VI, et al New environmental metabarcodes for analysing soil DNA: potential for studying past and present ecosystems. Mol Ecol 2012;21:1821–33. 10.1111/j.1365-294X.2012.05537.x 22486821

[45] Piñol J, Mir G, Gomez-Polo P, Agustí N. Universal and blocking primer mismatches limit the use of high-throughput DNA sequencing for the quantitative metabarcoding of arthropods. Mol Ecol Resour 2015;15:819–30. 10.1111/1755-0998.12355 25454249

[46] Vestheim H, Jarman SN. Blocking primers to enhance PCR amplification of rare sequences in mixed samples - a case study on prey DNA in Antarctic krill stomachs. Front Zool 2008;5:12 10.1186/1742-9994-5-12 18638418 PMC2517594

[47] Martin M. Cutadapt removes adapter sequences from high-throughput sequencing reads. EMBnet Journal 2011;17:10.

[48] Boyer F, Mercier C, Bonin A, Le Bras Y, Taberlet P, Coissac E. Obitools: a unix-inspired software package for DNA metabarcoding. Mol Ecol Resour 2016;16:176–82. 10.1111/1755-0998.12428 25959493

[49] Tedersoo L, Bahram M, Põlme S, Kõljalg U, Yorou NS, Wijesundera R, et al Global diversity and geography of soil fungi. Science 2014;346:1256688 10.1126/science.1256688 25430773

[50] Warinner C, Speller C, Collins MJ, Lewis CM Jr. Ancient human microbiomes. J Hum Evol 2015;79:125–36. 10.1016/j.jhevol.2014.10.016 25559298 PMC4312737

[51] Mann AE, Sabin S, Ziesemer K, Vågene ÅJ, Schroeder H, Ozga AT, et al Differential preservation of endogenous human and microbial DNA in dental calculus and dentin. Sci Rep 2018;8:9822 10.1038/s41598-018-28091-9 29959351 PMC6026117

[52] Taberlet P, Coissac E, Pompanon F, Gielly L, Miquel C, Valentini A, et al Power and limitations of the chloroplast trnL (UAA) intron for plant DNA barcoding. Nucleic Acids Res 2007;35:e14–e14. 10.1093/nar/gkl938 17169982 PMC1807943

[53] Riaz T, Shehzad W, Viari A, Pompanon F, Taberlet P, Coissac E. ecoPrimers: inference of new DNA barcode markers from whole genome sequence analysis. Nucleic Acids Res 2011;39:e145–e145. 10.1093/nar/gkr732 21930509 PMC3241669

[54] Valentini A, Taberlet P, Miaud C, Civade R, Herder J, Thomsen PF, et al Next‐generation monitoring of aquatic biodiversity using environmental DNA metabarcoding. Mol Ecol 2016;25:929–42. 10.1111/mec.13428 26479867

[55] Miyazaki Y. Nogyo zensyo [in Japanese]. 1697.

[56] Ehara A, Ishikawa N, Higashiyotsuyanagi S. Nihon shokumotsu-shi [in Japanese]. Yoshikawa Kobunkan; 2009.

[57] Tsutaya T, Nagaoka T, Kakinuma Y, Kondo O, Yoneda M. The diet of townspeople in the city of Edo: carbon and nitrogen stable isotope analyses of human skeletons from the Ikenohata-Shichikencho site. Anthropol Sci 2016;124:17–27.

[58] Darbyshire B, Henry RJ. Differences in fructan content and synthesis in some Allium species. New Phytol 1981;87:249–56.

[59] Shibutani A. The Present Issues on Starch Analysis in Japanese Archaeology [in Japanese]. Cultura Antiqua 2015;67:108–18.

[60] Iwatsuki K, Boufford DE, Ohba H. Flora of Japan: Pteridophyta and Gymnospermae. Tokyo: Kodansha; 1995.

[61] Makino T. New Makino’s illustrated flora of Japan [in Japanese]. Tokyo: Hokuryukan; 2008.

[62] Kitagawa M. Morisada-manko [in Japanese]. 1853.

[63] Yamato Honzo [in Japanese]. 1709.

[64] Hozumi H. Kyumin myoyaku-syu [in Japanese]. 1693.

[65] Dayanandan S, Ashton PS, Williams SM, Primack RB. Phylogeny of the tropical tree family Dipterocarpaceae based on nucleotide sequences of the chloroplast rbcL gene. Am J Bot 1999;86:1182 10449398

[66] Yonekura K, Kajita T. BG Plants Japanese-scientific names index (Ylist) 2003. http://ylist.info/index.html.

[67] Wakan Sansai Zue [in Japanese]. 1712.

[68] Kitamura N. Kiyu Shoran [in Japanese]. 1830.

[69] Lozano M, Subirà ME, Aparicio J, Lorenzo C, Gómez-Merino G. Toothpicking and periodontal disease in a Neanderthal specimen from Cova Foradà site (Valencia, Spain). PLoS One 2013;8:e76852 10.1371/journal.pone.0076852 24146934 PMC3797767

[70] Buckley S, Usai D, Jakob T, Radini A, Hardy K. Dental calculus reveals unique insights into food items, cooking and plant processing in prehistoric central Sudan. PLoS One 2014;9:e100808 10.1371/journal.pone.0100808 25028938 PMC4100759

[71] Cummings LS, Yost C, Sołtysiak A. Plant microfossils in human dental calculus from Nemrik 9, a Pre-Pottery Neolithic site in Northern Iraq. Archaeol Anthropol Sci 2016;40:118–9.

[72] Port JA, O’Donnell JL, Romero-Maraccini OC, Leary PR, Litvin SY, Nickols KJ, et al Assessing vertebrate biodiversity in a kelp forest ecosystem using environmental DNA. Mol Ecol 2016;25:527–41. 10.1111/mec.13481 26586544 PMC4737306

[73] Radini A, Nikita E, Buckley S, Copeland L, Hardy K. Beyond food: The multiple pathways for inclusion of materials into ancient dental calculus. Am J Phys Anthropol 2017;162:71–83. 10.1002/ajpa.23147 28105717

[74] Hardy K, Buckley S, Collins MJ, Estalrrich A, Brothwell D, Copeland L, et al Neanderthal medics? Evidence for food, cooking, and medicinal plants entrapped in dental calculus. Naturwissenschaften 2012;99:617–26. 10.1007/s00114-012-0942-0 22806252

[75] Sawafuji R, Cappellini E, Fotakis AK, Jersie-Christensen RR, Olsen JV, Hirata K. Proteomic profiling of archaeological human bone. Royal Society Open Science 2017;4:161004 10.1098/rsos.161004 28680659 PMC5493901

[76] Santana-Sagredo F, Lee-Thorp JA, Schulting R, Uribe M. Isotopic evidence for divergent diets and mobility patterns in the Atacama Desert, northern Chile, during the Late Intermediate Period (AD 900-1450). Am J Phys Anthropol 2014;156:374–87. 10.1002/ajpa.22663 25385676

[77] Tsutaya T. Post-weaning diet in archaeological human populations: A meta-analysis of carbon and nitrogen stable isotope ratios of child skeletons. Am J Phys Anthropol 2017;164:546–57. 10.1002/ajpa.23295 28786488

[78] Hardy K, Blakeney T, Les Copeland, Kirkham J, Wrangham R, Collins M. Starch granules, dental calculus and new perspectives on ancient diet. J Archaeol Sci 2009;36:248–55.

[79] Zhang N, Dong G, Yang X, Zuo X, Kang L, Ren L, et al Diet reconstructed from an analysis of plant microfossils in human dental calculus from the Bronze Age site of Shilinggang, southwestern China. J Archaeol Sci 2017;83:41–8.

[80] Pansu J, Giguet-Covex C, Ficetola GF, Gielly L, Boyer F, Zinger L, et al Reconstructing long-term human impacts on plant communities: an ecological approach based on lake sediment DNA. Mol Ecol 2015;24:1485–98. 10.1111/mec.13136 25735209

[81] Alsos IG, Sjögren P, Edwards ME, Landvik JY, Gielly L, Forwick M, et al Sedimentary ancient DNA from Lake Skartjørna, Svalbard: Assessing the resilience of arctic flora to Holocene climate change. Holocene 2016;26:627–42.

[82] Pedersen MW, Overballe-Petersen SR, Ermini L, Sarkissian CD, Haile J, Hellstrom M, et al Ancient and modern environmental DNA. Philos Trans R Soc Lond B Biol Sci 2014;370:20130383–20130311. 10.1098/rstb.2013.0383 PMC4275890 25487334

[83] Nistelberger HM, Smith O, Wales N, Star B, Boessenkool S. The efficacy of high-throughput sequencing and target enrichment on charred archaeobotanical remains. Sci Rep 2016:1–11. 10.1038/s41598-016-0001-8 27881876 PMC5121605

