# Supplementary figures and images for: Ancient DNA analysis of food remains in human dental calculus from the Edo period, Japan
Source: PLoS One. 2020 Mar 4;15(3):e0226654. doi: 10.1371/journal.pone.0226654 (PMC7055813; doi:10.1371/journal.pone.0226654)

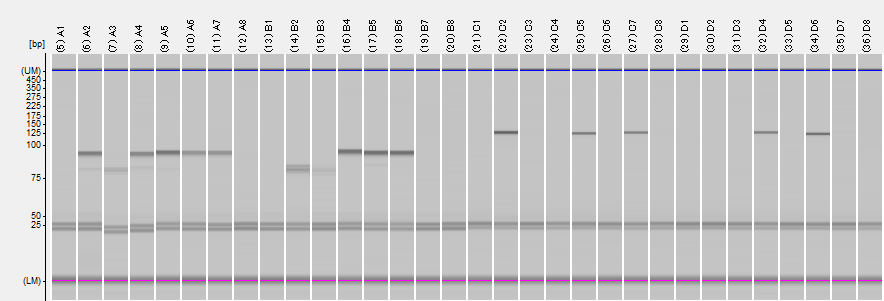

Supplement: S1 Fig — (TIF) [file pone.0226654.s005.tif]
